# Electrophysiological and Behavioral Responses of the Whitestriped Longhorned Beetle, *Batocera lineolata*, to the Diurnal Rhythm of Host Plant Volatiles of Holly, *Viburnum awabuki*

**DOI:** 10.1673/031.013.8501

**Published:** 2013-09-17

**Authors:** Hua Yang, Wei Yang, Chun-Ping Yang, Tian-Hui Zhu, Qiong Huang, Shan Han, Jiu-Jin Xiao

**Affiliations:** 1 Key Laboratory of Ecological Forestry Engineering of Sichuan Province, College of Forestry, Sichuan Agricultural University, Ya'an, Sichuan 625014, China; 2 Resource Protection and Ecotourism Department, College of Tourism, Sichuan Agricultural University, Dujiangyan, Sichuan 611830, China

**Keywords:** electroantennogram response, physiological condition

## Abstract

The adsorption method of Tenax-TA absorbent with GC-MS was used to analyze diurnal rhythms of volatiles from undamaged holly plants, *Viburnum awabuki* Kock (Dipsacales: Adoxaceae) holly infested by the white-striped longhorned beetle, *Batocera lineolata* Chevrolat (Coleoptera: Cerambycidae). Electroantennography and a Y-tube olfactometer were used to compare and analyze electroantennogram and behavioral responses of unmated male and female adults to the volatiles from *V. awabuki* (both undamaged and infested plants). The results of the GC-MS analysis showed that phytosterol and alkane are major volatiles for *V. awabuki*. The relative content of *V. awabuki* volatiles changed during the day. Electroantennogram and behavioral responses of unmated male and female adults to the volatiles from both undamaged and infested plants of *V. awabuki* were stronger between 08:00 and 10:00 and 16:00 and 18:00, which is consistent with early morning and evening feeding behaviors of adults in the field.

## Introduction

The white-striped longhorned beetle, *Batocera lineolata* Chevrolat (Coleoptera: Cerambycidae), is a polyphagous wood-boring insect that feeds on more than 20 plant species belonging to taxonomically distant plant families (Salicaceae, Juglandaceae, Fagaceae, Rosaceae, Caprifoliaceae, Betuleae, Oleaceae, Moraceae, Euphorbiaceae) (Sun and Zhao 1991; Gao et al. 1995; Li et al. 2008a, 2009a; Liang et al. 2008;). *B. lineolata* is among the most important wood-boring pests on poplar and walnut in Southern and Central China (Mei et al. 1998; Luo et al. 2000; Chen and Luo 2001) and can also damage white wax, birch, olive, rose, and holly (Xiao et al. 2003; Yang et al. 2010). *B. lineolata* is mainly distributed in China, Vietnam, Japan, India, and Myanmar (Chen et al. 1959). This species has one generation every 2–3 years and overwinters as larvae and adults in trunks (Yu et al. 2007). Overwintered adults exit their holes and feed on fresh bark at the end of April (Xia et al. 2005). Holly, *Viburnum awabuki* Kock (Dipsacales: Adoxaceae), is considered to be the preferred food for adults (Liang et al. 2008; Yang et al. 2011).

The larvae of *B. lineolata* damage the host plant, their borings criss-crossing within the trunk, and cause the eventual death of whole plants, which leads to a loss of commercial timber value (Li et al. 2009b). Currently, biological (nematode, parasitoid, and white mucardine fungi) and chemical (spraying with pyrethroids, organophosphates, and phenylpyrazoles) measures are applied to control *B. lineolata* larvae, but both methods are difficult and relatively ineffective (Zhu et al. 1995; Lu et al. 1996; Xiao et al. 2003; Li et al. 2008b). When *B. lineolata* adults emerge from their holes, they demonstrate a unique and obvious behavioral orientation (Liang et al. 2008), i.e., a tendency to move toward the preferred plant. Thus, it may be possible to develop new and effective control measures based on the method of detection of plants by *B. lineolata* adults.

Many phytophagous insects use odors as cues for orientation to food resources, either for their nutrition, mate location, or oviposition (Stanjek et al. 1997; Ruther et al. 2000; Inui et al. 2003; van Der Goes et al. 2006; Hu et al. 2009). Plants synthesize and release blends of volatile organic compounds (VOCs). Many plant VOCs have been identified, and these kairomones include fatty acid derivatives, phenyl-propanoids, and isoprenoids, among others (Visser 1986; Bernays and Graham 1988; Bruce et al. 2005). The plant VOCs released vary with factors such as age, physiological condition, diurnal rhythm, season, microhabitat, and environment (Lu et al. 2003). *V. awabuki* has been used as a trapping crop to lure *B. lineolata* adults in China (Liang et al. 2008; Yang et al. 2010, 2011), but the differences between undamaged and infested *V. awabuki* were unknown. Furthermore, when VOCs gave the strongest lure to *B. lineolata* during 1 day was also unknown.

In this study volatile chemical composition and amounts were compared between undamaged and infested *V. awabuki* branches, and electrophysiological and behavioral responses of *B. lineolata* to volatiles under different physiological conditions were assessed. This work may lead to the identification of plantbased attractants to be used in control and monitoring strategies for *B. lineolata*.

## Materials and Methods

### Insects

*B. lineolata* adults were collected in fields near Luojiang, China, during April and May 2010. The adult beetles had just finished their emergence and had not mated. Female and male beetles were held in separate cages (60 cm × 60 cm × 60 cm, stainless steel mesh) at room temperature (25 ± 2° C) until the experiment. They were fed with fresh bark of *V. awabuki*. The characteristics used to identify mated *B. lineolata* were the villi on the abdomen of mated males and the obvious mating plaques on the backside of the mated females (Ji et al. 1996).

### Plants

*V. awabuki* (1.5 m high and 2 cm DBH) taken from poplar woods in Luojiang County, Deyang City, Sichuan Province, were placed in pots (one plant per pot) in March 2010. The plants were watered once every 2 days and grew at room temperature. Potted *V. awabuki* were placed into the breeding cages (one pot per cage). After which placing the plants in the cages, 4 pairs of *B. lineolata* were allowed to feed on the plant for 24 hours. Beetles and their waste were removed before volatile extraction. Intact, undamaged plants were used for comparison.

### Extraction and identification of volatiles

A polyester cooking bag (355 mm × 508 mm; Reynolds, www.reynoldskitchens.com)equipped with 2 glass tubes in a row, a charcoal filter tube, and a Tenax-TA tube (50 mg absorbent, 60–80 mesh; glass outer diameter: 6 mm; length: 85 mm; Supelco, www.sigmaaldrich.com) were used to cover *V*. awabuki. Air was pumped in by a pocket pump QC-2B (Beijing Labor Protection Research Institute, China) from the charcoal filter tube to the polyester cooking bag, and then pumped into the Tenax-TA tube at 0.5 L/min. VOCs were collected for 1 hr at room temperature. Then, 5 mL of steamed n-hexane(chemically pure by Chengdu Kelong Chemical Reagent Factory, www.cdkelong.com) was used repeatedly to clean the Tenax-TA tube, and the solution was placed into the sample bottle of 8 mL. Next, N2 was used to concentrate the solution by 2 mL, and the solution was then stored in an ultra cold freezer (Sanyo, www.panasonic.net/sanyo) and used for volatile identification, electroantennogram (EAG), and behavioral measurement. One sample of the volatile of undamaged and infested *V. awabuki* was taken every 2 hours from 08:00 to 18:00.

Extracts were analyzed by GC-MS using a Shimadzu gas chromatograph (model 17A, www.shimadzu.com) coupled to a Shimadzu QP5050A electron ionization mass detector. The GC was operated in the splitless mode and was equipped with a DB-5 capillary column (30 m × 0.25 mm × 0.25 µm) (Agilent Technologies, www.agilent.com). The column's oven temperature was programmed to rise from an initial temperature of 40° C (3 min) to 220° C at 5° C per minute and then to 250° C at 8° C per minute. The temperature was then maintained for 5 min. In the mass spectrometer, the electron impact ion source voltage was 70 eV. The temperature for the GC/MS connector was 250° C, and the temperature of the ion source was 200° C. The scanning speed was 0.4 sec, and the scanning range was 40–450 m/z. The electric current in the filament was 150 µA. The mass spectrogram was checked by the standard mass spectrogram from the NIST database in this device, and the relative materials were consulted (Hao and Ha 2000; Zhao et al. 2001) to determine the chemical component.

### Electroantennogram experiments

Recordings of the responses of female and male *B. lineolata* antennae to plant VOCs were made using Syntech (www.syntech.nl)equipment comprising micromanipulators, a CS-05 stimulus air controller, and an IDAC signal connection box for data acquisition. EAG signals and data were analyzed using a customized software package (EAG for Windows XP; Syntech. The antennae of *B. lineolata* were excised and mounted between Ag and AgCl glass electrodes filled with Ringer solution (Roelofs 1984; Teodora et al. 2010).

A 2 µL odor source sample was taken with a micro-sampler. It was uniformly dripped onto folded filter paper (1.5 × 1.5 cm) that was put into a 10 cm sample tube. The end of the sample tube was connected to the odor stimulating control device. When the baseline was stable, the antenna was stimulated. The stimulation time was 0.5 sec, and the interval between stimulations was 30 sec, which permitted recovery of the antennal receptors. For each compound, 6 antennae (from 6 different adults) were tested, and each antenna was stimulated 5 times. Distilled hexane was used as the standard, and the mean of the observed value for each sample was divided by the mean of the 2 standard values to give the relative value of antennal responses.

### Bioassay of the behavioral response

A glass Y-tube olfactometer with an inside diameter of 15 cm was used to conduct the bioassays. The main arm of the device was 30 cm long, and the 2 side arms were 25 cm long. The angle between the 2 side arms was 75°, and the ends of the arms had ground-glass edges. The 2 side arms were connected to 2 250 mL volumetric flasks by Teflon tubes. A micro-sampler was used to extract 10 µL VOCs and solution (distilled hexane) for the comparison respectively, and then each was dropped on filter papers (1 × 1 cm). Filter papers were put into the 2 volumetric flasks separately. The volumetric flasks were connected by Teflon tubes to a distilled water humidification bottle and a charcoal filter. The air flow speed was controlled at 0.5–…0.6 L/min. Tests were run 08:00–12:00 when the temperature of the laboratory was 25 ± 2° C. *B. lineolata* adults were introduced into the inlet of the main arm of the Y-tube, and timing began after they had moved forward 10 cm from the inlet. Adults had to make a choice at the junction of the Y-tube to 1 of the side arms. Tests were conducted for 5 minutes to observe each *B. lineolata* adult. If an insect went forward 10 cm into a side arm and stayed for at least 1 minute, it was recorded as having made a choice of this odor; otherwise, it was recorded to have made no choice. After every 5 adults, the 2 arms of the Y-tube were interchanged to eliminate the possible influence of the different arms on the behavior of *B. lineolata* adults. When each treatment was finished, the Y-tube olfactometer, Teflon tubes, and volumetric flasks were washed with alcohol and allowed to air dry. Each treatment was performed for 30 *B. lineolata* adults in the same eclosion time, and each *B. lineolata* adult was used only once.

### Statistical analyses

Statistical analyses was performed using the SPSS10.0 statistical package (SPSS Inc, IBM, www.ibm.com). A one-factor randomized complete block ANOVA was conducted on the EAG data and the relative content of VOCs. Fisher's protected least significant difference LSD multiple comparison procedure was used in EAG responses of unmated male and female adults to VOCs from *V. awabuki* and relative content change of *V. awabuki* VOCs at different times (Dong et al. 2000; Liu et al. 2005). A Chi-square test was used to compare the rate of attraction (Sokal and Rohlf 1995). The rate of attraction was calculated according to the following formulas (Ding et al. 1996; Yan et al. 2006):

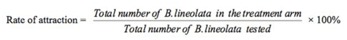



## Results

### Comparison of compositions of VOCs from undamaged and infested *V. awabuki*

**Figure 1.**
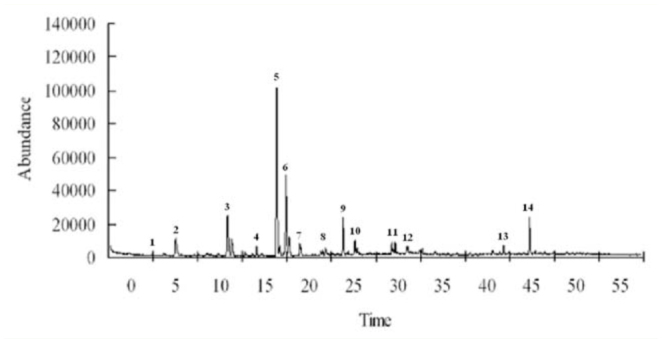
GC-MS analyses of volatiles from undamaged *Viburnum awabuki*. The 14 compounds are: benzyl alcohol (1), decane (2), nonanal (3), tridecane (4), limonene (5), 3-methyl-butanoic acid (6), decanal (7), isohexyl alcohol (8), hexadecane (9), isobutyl phthalate (10), butanamide (11), 2-methyloctane (12), butyl acrylate (13), and (Z)-3-hexen-l-ol (14). High quality figures are available online.

**Figure 2.**
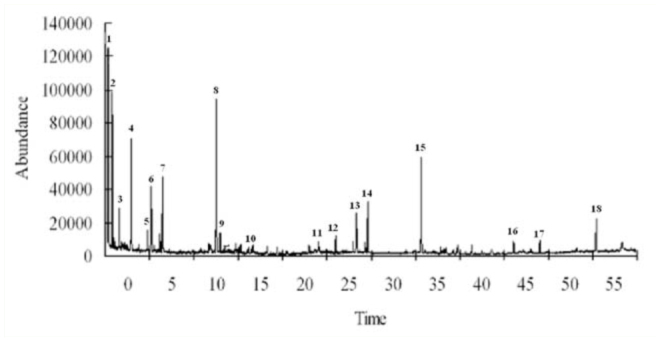
GC-MS analyses of volatiles from infested *Viburnum awabuki*. The 18 compounds are permethyl 99A (1), octyl alcohol (2), nonanal (3), cyclooctatetraene (4), limonene (5), decanal (6), tridecane (7), 3-methyl-butanoic acid (8), butyl acrylate (9), heptyl chloroacetate (10), hexanal (11), isobutyl phthalate (12), 2-bromo-octane (13), butanamide (14), (Z)-3-hexen-1-ol (15), 3,4,5,6-tetramethyloctane (16), indole (17), and hexadecane (18). High quality figures are available online.

The results of GC-MS analysis showed that phytosterols such as benzyl alcohol, octanol, and isohexyl alcohol, and alkanes such as decane, tridecane, hexadecane, and 2-methyl octane were major VOCs for *V. awabuki*. There were 14 compounds in undamaged plants (Figure 1) and 18 compounds in plants infested by *B. lineolata* (Figure 2).

### Daily changes in relative contents of *V. awabuki* VOCs

**Figure 3.**
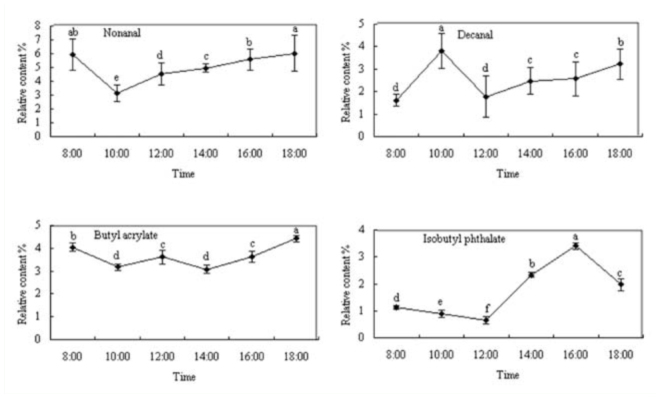
Daily dynamics of 4 volatile chemicals from undamaged *Viburnum awabuki*. Figures marked with the same lowercase letter were not significantly different (ANOVA followed by LSD, *p* < 0.05). Error bars ± SE (n = 3). High quality figures are available online.

For relative contents of *V. awabuki* VOCs from undamaged hosts, there was an obvious daily change in nonanal, decanal, butyl acrylate, and diisobutyl phthalate (Figure 3). Relative peak content of nonanal and butyl acrylate appeared at 18:00, while peak content of decanal appeared at 10:00 and peak content of diisobutyl phthalate appeared at 16:00.

**Figure 4.**
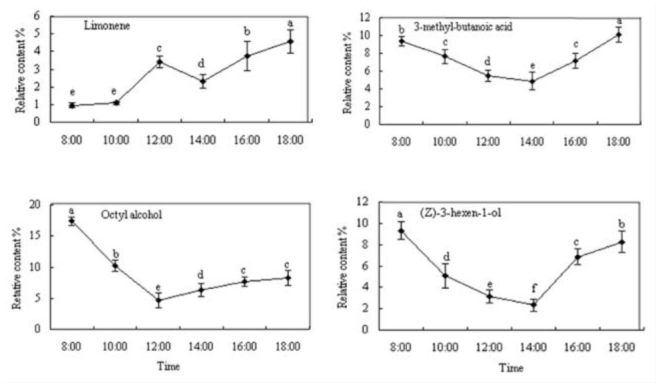
Daily dynamics of 4 volatile chemicals from infested *Viburnum awabuki*. Figures marked with the same lowercase letter were not significantly (ANOVA followed by LSD, *p* < 0.05). Error bars ± SE (n = 3). High quality figures are available online.

For relative contents of *V. awabuki* VOCs from infested hosts, there was an obvious daily change in limonene, 3-butyric acid methyl, octanol, and (Z)-3-hexene-1-alcohol (Figure 4). The relative peak content of limonene and 3-butyric acid methyl appeared at 18:00, while the peak content of octanol and (Z)-3-hexene1-alcohol appeared at 08:00.

### EAG response of *B. lineolata* adults to *V. awabuki* VOCs at different times

**Figure 5.**
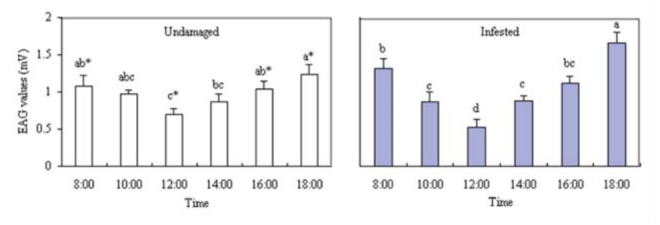
EAG responses of *Batocera lineolata* unmated females to VOCs from *Viburnum awabuki* at different times. White bars (undamaged plants) marked with the same lowercase letter were not significantly different. Grey bars (infested plants) marked with the same lowercase letter were not significantly different (ANOVA followed by LSD, *p* < 0.05). Asterisks signify a significant difference between undamaged and infested plants (*t*-test, *p* < 0.05). Error bars ± SE (n = 6). High quality figures are available online.

In order to evaluate the electrophysiological activity, VOCs in different periods were tested for the EAG responses they elicited in *B. lineolata* antennae. The highest EAG responses of unmated females to VOCs from undamagedand infested plants occurred at 18:00 (*p* >0.05; Figure 5). For the 2 different physiology states of *V. awabuki*, there was a significant difference among EAG responses of unmated males at 08:00, 12:00, 16:00, and 18:00 (*p* < 0.05).

**Figure 6.**
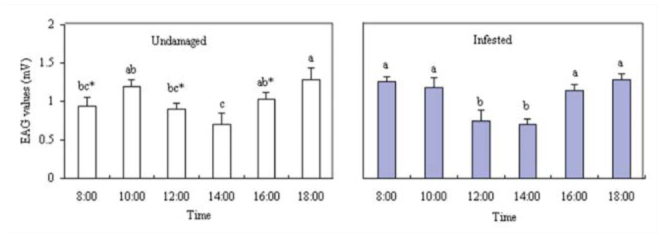
EAG responses of *Batocera lineolata* unmated males to VOCs from *Viburnum awabuki* at different times. White bars (undamaged plants) marked with the same lowercase letter were not significantly different. Grey bars (infested plants) marked with the same lowercase letter were not significantly different (ANOVA followed by LSD, *p* < 0.05). Asterisks signify a significant difference between undamaged and infested plants (*t*-test, *p* < 0.05). Error bars ± SE (n = 6). High quality figures are available online.

**Figure 7.**
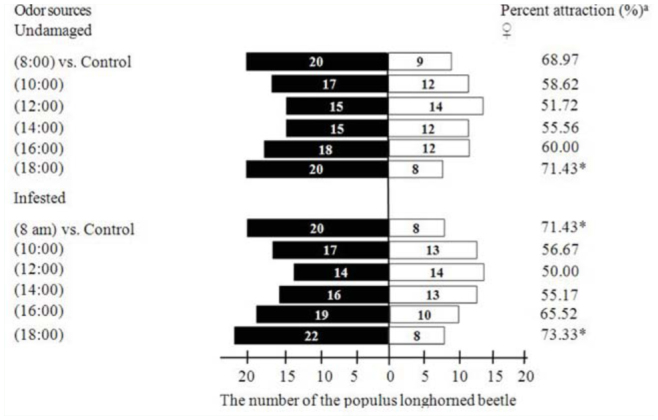
Behavioral response of *Batocera lineolata* unmated females to VOCs from *Viburnum awabuki* in different times. (White columns = control arms, black columns = treatment arms, *n* =30, when time more than 5 min, *B. lineolata* will not move toward control arms or treatment arms, which is regarded as “no response”). ^a^ *Asterisk* shows a significant rate of attraction (Chi-square test *p* < 0.05). High quality figures are available online.

There was no difference in EAG responses of unmated male to VOCs from infested plants at 08:00, 10:00, 16:00, and 18:00 (*p* > 0.05; Figure 6). The highest EAG responses of unmated males were at 18:00 (*p* > 0.05) from the undamaged plant. For the 2 different physiology states of *V. awabuki*, there was a significant difference among EAG responses of unmated males at 08:00, 12:00, 16:00, and 18:00 (*p* < 0.05).

### Laboratory bioassay of *B. lineolata* adults to *V. awabuki* VOCs at different times

There was a strong attraction (higher rate of attraction) of *V. awabuki* VOCs to unmated female *B. lineolata* at different times (Figure 7). The result of the Chi-square test showed that there was a significant attractive effect from undamaged volatiles (18:00) toward unmated female *B. lineolata* (χ^2^ = 4.32; df = 1; *p* < 0.05), and a significant attractive effect from infested hosts’ (08:00) (χ^2^ = 4.32; df= 1; *p* < 0.05) volatiles (18:00) (χ^2^= 5.63; df = 1; *p* < 0.05) toward unmated female *B. lineolata*.

**Figure 8.**
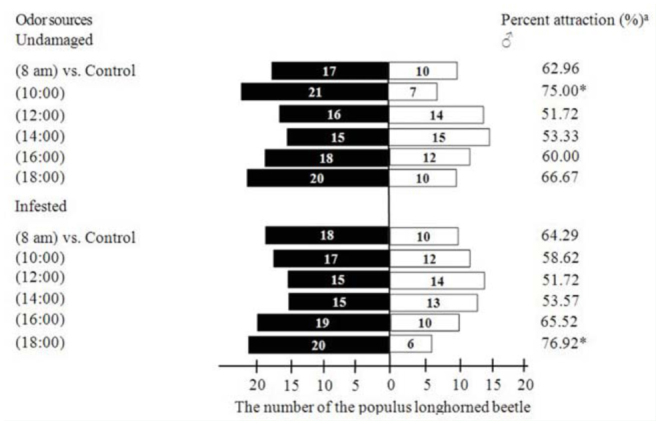
Behavioral response of *Batocera lineolata* unmated males to VOCs from *Viburnum awabuki* at different times. White columns = control arms, black columns = treatment arms, n = 30. When the time reached 5 min and the *B. lineolata* did not move toward the control or treatment arm it was regarded as “no response.” Asterisks signify a significant difference in rate of attraction (Chi-square test *p* < 0.05). High quality figures are available online.

The highest rate of attraction of *V. awabuki* VOCs to unmated male *B. lineolata* at 18:00 was nearly 77% (Figure 8). The results of the Chi-square test showed that there was a significant attractive effect of undamaged volatiles (10:00) (χ^2^= 6.04; df = 1; *p* < 0.05) and infested volatiles (18:00) (χ^2^= 6.50; df = 1; *p* < 0.05) toward unmated *B. lineolata* adults.

## Discussion

According to the results of GC-MS analysis, the two different *V. awabuki* states had different volatile compositions. After *V. awabuki* was used to feed *B. lineolata*, volatile composition changed and new compositions were found, such as permethyl 99A, octyl alcohol, cyclooctatetraene, heptyl chloroacetate, hexanal, 2-bromo-octane, 3,4,5,6tetramethyloctane, and indole. Research has shown that the type and quantity of volatiles released from plants bitten by phytophagous insects are different from normal volatiles (Mamiya and Enda 1972), and these new compositions are generated by the lure after the plant has been bitten by phytophagous insects (Degenhardt and Lincoln 2006). Paré and Tumlinson (1997) adopted CO_2_-impulse labeling to prove that those new compositions and VOCs that cannot be released by undamaged plants can be made after the plant has been bitten by phytophagous insects.

The relative content of nonanal, decanal, butyl acrylate, and diisobutyl phthalate released from undamaged *V. awabuki*, and limonene, 3-butyric acid methyl, octanol, and (Z)-3– hexene-1-alcohol from infested plants was different in 6 periods of time during the same day. According to the analysis of Loughrin et al. (1994), terpenoid content in the volatiles released from *Spodoptera exigua* has an obvious daily change. After Martin et al. (2003) took methyl jasmonate to spray on *Picea abies*, the induced volatile also had a rhythm of daily and nightly change. The research of Zhang et al. (1999) showed that monoterpene, sesquiterpene, and green volatiles from the branches of *Betula pendula* And *Sambucus williamsii* were influenced by temperature change during day time and increased with temperature rise within the range of 16–24° C, but there was no increase after the temperature rose above 24° C.

The EAG response of undamaged and infested plants of *V. awabuki* to unmated male and female *B. lineolata* was stronger between 08:00–10:00 and between16:00–18:00 than between 12:00–14:00. The behavior response results matched the EAG response results. It was found that the feeding behavior of plantfeeding insects matched the changes of certain components in host plants (Li et al. 2009). Yan et al. (1997) found that *B. lineolata* ate at dawn and dusk in the field, which matches the results of the EAG response test and the laboratory bioassay. Further study is required to determine how the circadian rhythm of the host plant affects the behavior of *B. lineolata*.

Plant volatiles induced by insects can effect the behavior selection of phytophagous insects and plays a role in chemical communication between individual plants (Visser 1986). Plant volatiles induced by insects are easily detectable to insects, and phytophagous insects can learn the status of host plants by the information in induced volatiles and perform corresponding behavior responses (Loughrin et al. 1995; Bolter et al. 1997; Dicke 1997). In our study, the EAG responses of unmated females to VOCs of infested plants were stronger at 08:00 and 18:00 than they were to undamaged plants at the same times, and the EAG responses for unmated males to VOCs of infested plants were stronger at 8:00 than they were to undamaged plants at the same time. Although there was no analysis of the difference between behavioral responses to VOCs from undamaged and infested plants, the lure rate matched the EAGs responses (Figures 7, 8), which means that VOCs released from infested *V. awabuki* had stronger attractiveness to *B. lineolata*.

VOCs could be utilized in developing traps for detecting and monitoring populations of *B. lineolata*. The behavioral role of other VOCs inducing relatively high electrophysiological activity on *B. lineolata* antennae needs to be studied in more detail.

## Abbreviations:

EAG,: electroantennogram;
VOC,: volatile organic compound
